# A time-resolved metabolomics study of potato cultivar responses to *Pseudomonas syringae*

**DOI:** 10.3389/fpls.2026.1760708

**Published:** 2026-02-11

**Authors:** Lindiwe Mahlangu, Khayalethu Ntushelo, Ntakadzeni Edwin Madala, Phumzile Sibisi

**Affiliations:** 1 Department of Agriculture and Animal Health, University of South Africa, Johannesburg, South Africa; 2 Department of Biochemistry and Microbiology, University of Venda, Thohoyandou, South Africa

**Keywords:** flavonoids, glycoalkaloids, lipid signalling, metabolomics, phenylpropanoid pathway, potato cultivars, *Pseudomonas syringae*, resistance biomarkers

## Abstract

**Introduction:**

The bacterial plant pathogen *Pseudomonas syringae* poses a threat to various crops, including potato (*Solanum tuberosum*). Its pathogenicity and virulence are enabled by, among other factors, its type III secretion system, which delivers effectors into the host plant. While differences in cultivar responses offer a sustainable control strategy, the underlying temporal metabolic mechanisms in potatoes remain poorly characterised.

**Methods:**

This study employed an untargeted LC-MS metabolomics to investigate dynamic metabolic reprogramming in contrasting potato cultivars, Sifra and Valor, following inoculation with *P. syringae*. The metabolomic analysis was conducted at three intervals: 24 hours, 48 hours, and 72 hours post-inoculation.

**Results:**

Our results revealed distinct, genotype-specific response kinetics. Neither cultivar exhibited significant pathogen-responsive metabolomic reprogramming at 24 hours post-inoculation. At 48 hours post-inoculation, levels of 14 metabolites were elevated in the inoculated Valor samples, while Sifra showed minimal reprogramming. The Valor responses were not sustained and had subsided at the 72 hour time point. Meanwhile, by 72 hours, a spike in 11 metabolites was observed in the inoculated Sifra, with almost no upregulation of these in Valor. Among the spiked metabolites in Valor and Sifra were metabolites with a proven role in pathogen response. In total, there were 5 overlapping heightened metabolites between Valor and Sifra.

**Discussion:**

While cultivars are expected to differ in their response to pathogen inoculation, resistance mechanisms and timing of response remain obscure and the present study uncovered varied time-dependent pathogen-induced metabolomic shifts of the two cultivars. This study added the much-needed knowledge of resistance responses with a new suite of response metabolites.

## 1 Introduction

Plant diseases present a significant and growing challenge to agricultural productivity and global food security (Velásquez et al., 2018). Among the economically important crops in the Solanaceae family, the potato (*Solanum tuberosum* L.) is a vital staple food source, yet it is particularly susceptible to various pathogens (Knapp et al., 2004; Platonov et al., 2024). These pathogens, which encompass bacteria, fungi, and viruses, drastically reduce both yield and quality (Jagatheeswari, 2014). The widespread cultivation of Solanaceae crops also aids in the spread of these pathogens across regions and continents (Galdino et al., 2015).

One of the principal bacterial threats is *Pseudomonas syringae*, a Gram-negative pathogen known to infect numerous plant species (Yang et al., 2023). It typically enters the plant through natural openings such as stomata and subsequently colonises the apoplast, leading to symptoms such as water-soaked lesions, chlorosis and eventually tissue death. Bacterial build-up, tissue colonisation and early disease progression hinder photosynthesis, and this results in moderate to severe yield losses (Katagiri et al., 2002; Jones and Wildermuth, 2011) depending on the extent of infection. *Pseudomonas syringae* is highly adaptable and often evades plant immune responses (Cai et al., 2011). Although its impact on tomatoes has been extensively studied, its infection dynamics and behaviour in potatoes are less well understood, despite the bacterium’s confirmed pathogenicity to this crop (Baltrus et al., 2017; Charkowski et al., 2020). Management of this pathogen is often hampered by its evasive nature and its ability to endure fluctuating environmental conditions and to propagate through rain splash, irrigation, and infected seeds or tools (Jones and Wildermuth, 2011). Conventional measures for control include copper-based bactericides, sanitation, and resistant varieties (Lindeberg et al., 2009). Bacterial pathogens are increasingly becoming resistant to copper-based products, and sanitation has limitations. Furthermore, the genetic variation within the *P. syringae* population complicates the establishment of lasting host resistance (Jones and Wildermuth, 2011). Despite this, host resistance remains a viable and sustainable management tool.

Plant defence relies heavily on rapid metabolic changes that facilitate the production of antimicrobial compounds, strengthen cell walls, and activate defence signalling pathways (Kaur et al., 2022). In this context, metabolomics has emerged as an essential tool for studying plant-pathogen interactions, as it provides a comprehensive view of the biochemical shifts that occur during infection (Sakurai, 2022). Different cultivars may respond to infection in distinct metabolic ways, likely accounting for the varying levels of response observed (Aversano et al., 2017; Mashabela et al., 2023). However, the role of these metabolic differences in the relationship between potatoes and *P. syringae* has not been thoroughly investigated. Furthermore, little is known about the timing of these metabolic defences, particularly regarding how quickly they commence and how long they are sustained. This timing could be crucial in determining whether the plant can effectively respond to infection (Askri et al., 2025).

Based on patterns observed in other plant-pathogen systems (Das et al., 2025), we hypothesized that some potato cultivars with more effective responses are likely to activate robust metabolic defences early after infection and maintain these responses over time. In contrast, cultivars with less effective responses may exhibit slower or short-lived reactions that are inadequate to limit infection. These variations in defence timing and intensity may help clarify the differing response patterns among cultivars.

This study presents a detailed, time-based metabolomic analysis of potato cultivars responses to *P. syringae* infection. We examined two cultivars: Valor, which displayed more effective early responses albeit short-lived, and Sifra, characterised by delayed responses. Our objectives were to identify the key metabolites and pathways associated with different response patterns, determine whether effective defence arises from pre-existing metabolic preparedness or rapid activation following infection, and uncover specific time-related metabolic signatures indicative of effective defence responses. Given that this is a new combination of experimental units, the findings provide new and unique insights into the biochemical mechanisms behind differential responses and highlight potential biomarkers for breeding potatoes with enhanced defensive capabilities against *P. syringae*.

## 2 Materials and methods

Two cultivars, Sifra and Valor, which have varying resistance to *Pseudomonas syringae* (strain BD2110) was selected for study. Sixteen plants of each cultivar were divided into two groups of eight. One group was inoculated, and the other was uninoculated. Leaf metabolomic analyses were conducted at 24, 48, and 72 hours after inoculation. Metabolomic profiles of the two cultivars were compared, as well as profiles of the different time points across the two cultivars.

### 2.1 Experimental site and conditions

The experiment was conducted at the Horticulture Research Centre, University of South Africa, located in Florida, South Africa. The plants were grown outdoors under summer conditions, with daytime temperatures ranging from 18°C to 28°C and nighttime temperatures between 15°C and 18°C. All plants were placed in mesh cages measuring 60 × 60 × 90 cm. Each mesh cage housed eight plants, which were replicates. Manual irrigation was applied as necessary throughout the study.

### 2.2 Plant material and leaf inoculation

Two commercially relevant potato cultivars, namely, Sifra and Valor, with contrasting responses to *P. syringae*, as identified in preliminary screenings, were selected. Certified disease-free tubers were directly sown into 20-cm pots filled with sterilised potting soil. Thirty-five days after sowing, when the plants had developed four fully expanded compound leaves, they were inoculated with a cell suspension of the bacteria. Inoculation was done following the method outlined by Djami-Tchatchou et al. (2019a). In brief, the cell suspension was pressure-infiltrated into the abaxial surface of the leaves using a needleless syringe, with infiltration conducted at three sites per leaf across three leaves per plant. Only lower leaves were selected for inoculation, and control plants were mock-inoculated with the buffer. Successful inoculation was marked by an extensive permeation of the inoculum beneath the leaf epidermis.

### 2.3 Experimental design

**Figure 1:**
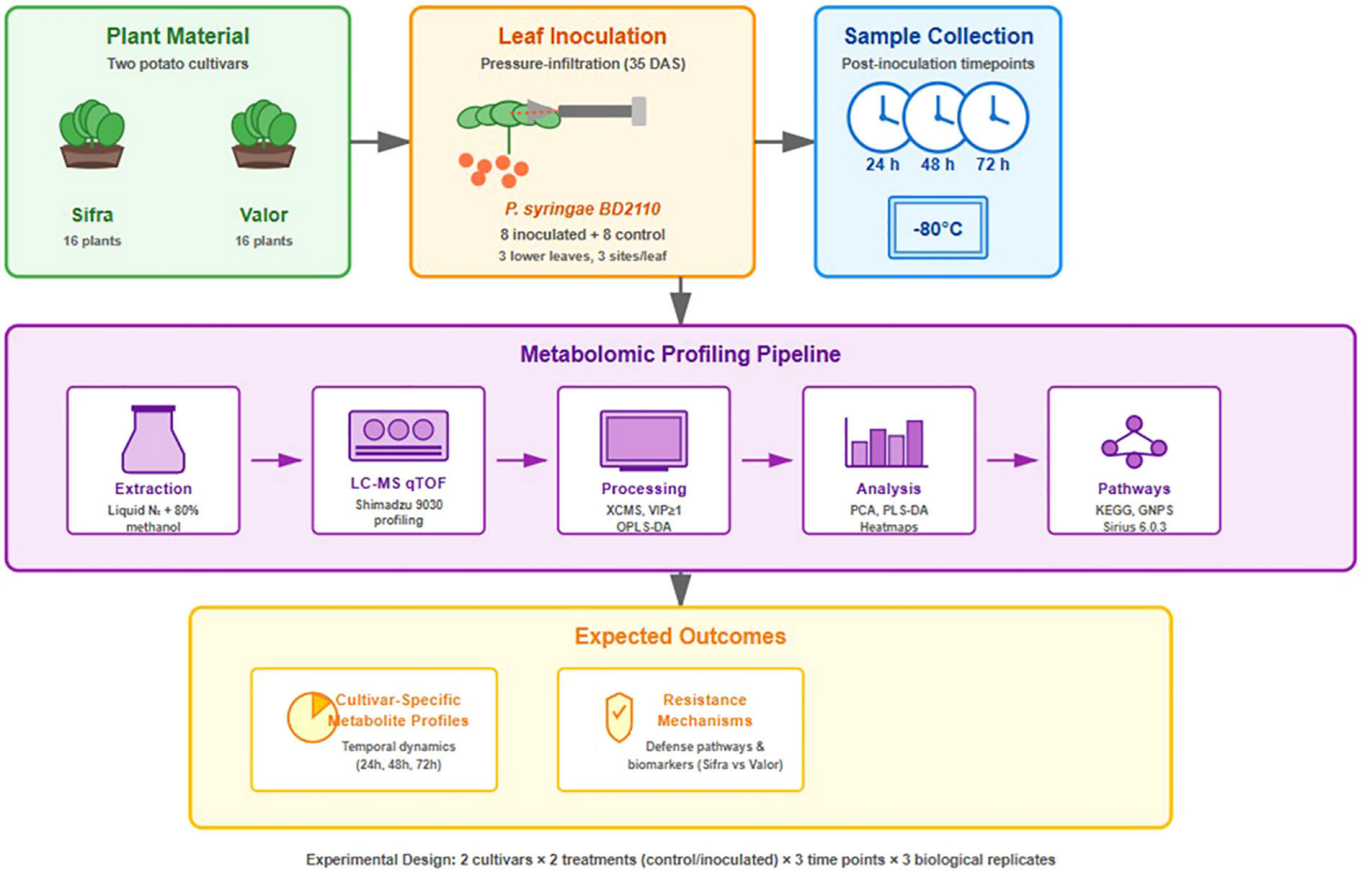
Experimental workflow for metabolomic analysis of potato cultivar response to *Pseudomonas syringae* pv. syringae infection. Two potato cultivars, Sifra and Valor (n=32) For metabolomic analysis, three samples per cultivar per treatment were collected at 24, 48, and 72 hours post-inoculation, extracted using 80% methanol, and analysed by LC-MS qTOF (Shimadzu 9030). Data analysis included OPLS-DA (VIP >1), PCA/PLS-DA (MetaboAnalyst, SIMCA®17), and metabolite identification using the GNPS, Sirius, HMDB, and KEGG databases to compare the differential metabolic responses between the cultivars.

The experiment included a total of 32 plants, with 16 plants assigned to each cultivar. Each cultivar group was further divided into control and treatment groups, resulting in four groups: control Sifra, treated Sifra, control Valor, and treated Valor. Each group consisted of eight plants. This design enabled a comparison of the differential responses of Sifra and Valor under pathogen stress conditions **Figure 1**.

### 2.4 Sample collection and metabolomic profiling

Leaf samples were collected at 24, 48, and 72 hours after inoculation from three randomly selected plants per treatment group. The collected samples were immediately frozen at -80°C and kept in the freezer until use. The frozen leaf tissue was ground in liquid nitrogen using a mortar and pestle, and secondary metabolites were extracted with methanol.

Metabolite extraction was performed according to a modified method from Madala et al. (2014). 1g of each leaf sample was ground into powder using liquid nitrogen in a mortar and pestle. The powdered samples were then weighed into 2 mL centrifuge tubes, to which 1.5 mL of 80% ice-cold methanol was added. The samples were vortexed for 30 seconds and subsequently sonicated for 30 minutes to enhance cell disruption. After sonication, the samples were centrifuged at 13, 950 rpm for 5 minutes at 4°C. The resulting supernatant was filtered through 0.22 µm nylon filters into glass vials with 500 µL inserts (Agela Technologies, Tianjin, China). Additionally, the extract was transferred into new 2 mL Eppendorf tubes and stored at 4°C until analysis. Three biological replicates were prepared for each group to ensure reproducibility.

Metabolite profiling was performed using a Shimadzu 9030 liquid chromatography–quadrupole time-of-flight mass spectrometer (LC-MS qTOF). Data from LC-MS included retention times, which indicate the duration each metabolite took to pass through the chromatography column; mass spectra, which provide information on molecular weight and structure; and quantitative data on metabolite abundance, derived from peak intensities. These data were exported in mzML format and pre-processed using XCMS Online (version 2.7.2), which incorporates XCMS (version 1.47.3) and CAMERA (version 1.34.0). The pre-processing employed UPLC-qTOF parameters with the centWave feature detection method. The maximum allowed m/z deviation was set at 15 ppm, the signal-to-noise ratio at 6, the peak width range at 5–20 s, the m/z difference at 0.01, and the pre-filters for peak number and intensity at 3 and 100. Individual samples yielded between 969 and 9, 749 features per file across six genotypes under control and bacterial treatments. Retention time correction was performed using the obiwarp method with a profStep of 1, while peak alignment and grouping were conducted using the density method, with a bandwidth of 5, an m/z width of 0.015, a minimum fraction of 0.5 across all samples, and a minimum sample requirement of 1. Missing peak intensities were filled using the Fill Peaks function. After these pre-processing steps, a Kruskal–Wallis non-parametric statistical test was performed, accompanied by *post-hoc* analysis, with a significance threshold of p ≤ 0.01. Following statistical filtering, 603 features were retained. From the filtered features, 15–16 metabolites were confidently annotated per time point (24, 48, and 72 hours post-inoculation). The retained features were further annotated and identified using, GNPS, and Sirius 6.0.3, referencing databases such as HMDB, MassBank, and PubChem. Metabolites were identified based on accurate mass, fragmentation patterns, and elemental composition. The data were then normalized, log-transformed, and Pareto-scaled, with missing values estimated before multivariate analysis. Principal Component Analysis (PCA), Partial Least Squares Discriminant Analysis (PLS-DA), and Orthogonal Partial Least Squares Discriminant Analysis (OPLS-DA) were conducted using SIMCA^®^ 17 software, with metabolites exhibiting Variable Importance in Projection (VIP) scores ≥1 considered significant. Additional data exploration included heatmap generation and pathway analysis using MetaboAnalyst, and the identified metabolites were mapped to biological functions using the Kyoto Encyclopedia of Genes and Genomes (KEGG).

## 3 Results

Cultivars Sifra and Valor displayed genotype-specific metabolomic responses to inoculation with *Pseudomonas syringae* pv*. syringae*. Additionally, these responses were time-dependent as they were differentiated at 24, 48 and 72 hours post-inoculation. Both cultivars, Valor and Sifra, exhibited minimal visible infection symptoms during the 72-hour assessment period following inoculation. The lack of visible symptoms at 72 hours post-inoculation is typical of *P. syringae* infection kinetics, as symptoms such as water-soaked lesions and chlorosis usually develop 5 to 7 days after inoculation (Katagiri et al., 2002; Cai et al., 2011). The 72-hour timeframe was intentionally chosen to observe early, pre-symptomatic metabolic defense responses. This period represents a crucial window during which plants activate their resistance mechanisms before any visible signs of disease appear (Ahuja et al., 2012; Bednarek, 2012). This strategy is consistent with established principles in plant defense metabolomics, as early metabolic signatures tend to be more predictive of eventual resistance outcomes compared to measurements taken during later stages of symptom development (Kumari et al., 2024; Das et al., 2025). This was expected, as symptom development requires a longer period. Therefore, the focus of the current work was limited to metabolite changes, which have been proven to occur as early as 24 hours post-inoculation with a pathogenic bacterium (Djami-Tchatchou and Ntushelo, 2017, 2019a, b). By examining metabolic reprogramming before symptom onset, it was aimed to differentiate active defense responses from secondary metabolic changes related to tissue damage and necrosis that occur in the later stages of infection.

### 3.1 Multivariate data analysis

Principal component analysis (PCA) was employed to investigate patterns in metabolite profiles of cultivars Sifra and Valor across various time points (24, 48, and 72 hours). PCA plots illustrate how samples cluster based on their overall metabolic composition, and in the present study, they emphasized variation attributed to cultivar differences and treatment duration.

**Figure 2:**
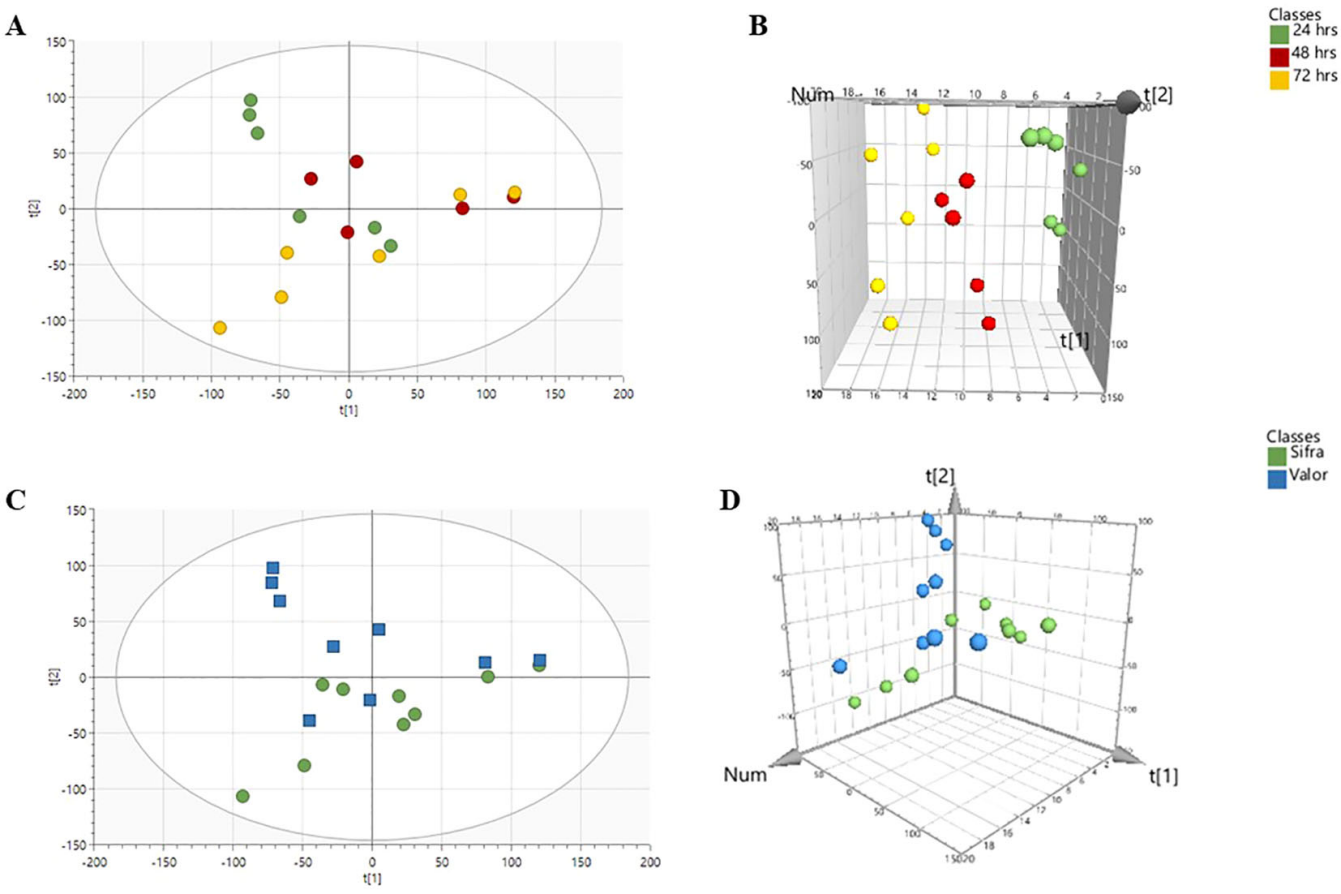
PCA score plots displaying the separation of metabolite profiles across different time points and cultivars. **(A, B)** 2D and 3D score plots illustrate the separation of samples based on time after treatment (24 h, 48 h, 72 h). **(C, D)** 2D and 3D score plots portray the separation between cultivars Sifra and Valor. The distinct clustering indicates that metabolite profiles are influenced by both treatment time and cultivar.

Focusing only on the duration of treatment, the PCA plots revealed clear distinctions between the sampling times with 24, 48 and 72 hours samples forming three distinct clusters (**Figures 2A, B**). On close examination, it was evident that the metabolite profile captured at 48 hours was transitional between 24 hours and 72 hours since it was between these two end points. The separation is clearer on the three-dimensional PCA plot (**Figure 2B**) as each time point occupied a unique region in the principal component space.

On cultivar-specific metabolite profiles (**Figures 2C, D**), the PCA plots showed a chromatographic separation between the two genotypes across all time points. The two cultivars formed clusters, however, with overlap, indicating that genetic background is a significant factor influencing metabolic composition, albeit without a strong expression characterised by non-overlapping PCA clusters. The tight clustering within each cultivar group reflected consistent metabolic profiles within genotypes.

This consistent separation implies that each cultivar possesses a unique baseline metabolic profile and potentially distinct responses to treatment. These findings suggest that genotype-specific regulation of metabolic pathways is crucial in shaping the overall metabolic landscape.

The PCA results indicated that both treatment time and plant genotype have a significant impact on metabolic profiles. The principal components capture the main sources of metabolic variation, with both genotype and time playing a significant role. Time influences the progression of the metabolic response, while genotype defines both the baseline and potentially the specific response mechanisms. This analysis underscores the importance of considering both time-related and genetic factors when interpreting differences and changes in plant metabolism.

**Figure 3:**
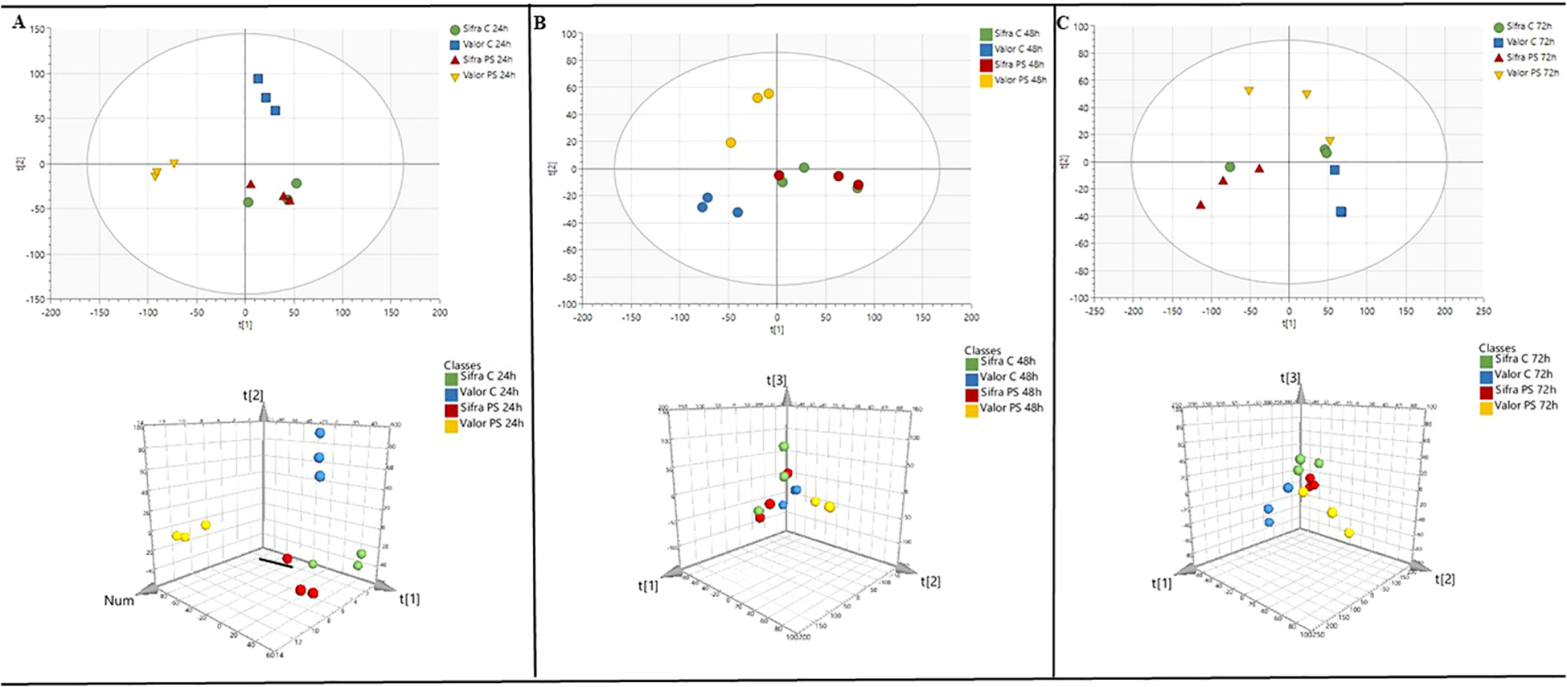
Partial least squares discriminant analysis (PLS-DA) of metabolite profiles in Valor and Sifra at **(A)** 24 h, **(B)** 48 h, and **(C)** 72 h post-treatment. Valor exhibited clear separation between treated and control groups, indicating a robust time-dependent metabolic response. In contrast, Sifra displayed minimal group separation, suggesting a more resistant metabolic response to treatment.

PLS-DA was further employed to investigate the metabolic response to treatment in the two potato genotypes, Valor and Sifra, at three time points, 24, 48, and 72 hours. This supervised method offers clearer group separation than PCA by incorporating information about treatment groups, allowing for a more focused view of how metabolite profiles differ in response to treatment over time (**Figures 3A–C**).

In both cultivars, *P. syringae*-treated samples and the control exhibited varied levels of separation from 24 hours through 72 hours. At 24 hours, the groups were already and most clearly distinct. The separation was especially greater in Valor, which indicates a strong and progressive shift in Valor’s metabolic profile following treatment. The results suggest that Valor undergoes extensive metabolic reprogramming in response to inoculation with *P. syringae*, reflecting a strongly pronounced metabolomic response within the first 24 hours of inoculation (**Figure 3A**).

In contrast, Sifra had less distinction between the *P. syringae*-treated and control groups, especially at 24 hours (**Figure 3A**). This limited separation suggests that the *P. syringae* had a much weaker impact on Sifra’s metabolite profile which carried over to the 48 and 72 hour time points (**Figures 3B, C**). The overall stability of Sifra’s metabolic profile across the time points may indicate that it is less responsive to the inoculation or that it has a more stable metabolic architecture under the tested conditions. Interestingly, the Valor samples are also less resolved at the 48 and 72 hour time points which may mean lessening of effects after the 24 hour time point.

Together, these results highlighted a clear genotype-dependent difference in metabolic responsiveness. Valor demonstrated a strong, time-dependent response to treatment, while Sifra exhibited a more stable and less altered profile. This suggests that Sifra may possess greater metabolic stability or tolerance to the treatment, potentially reflecting underlying genetic or physiological differences in stress perception or adaptation.

To enhance the distinction between treatment groups and eliminate unrelated orthogonal variation, OPLS-DA was applied at each time point. This method separates predictive variation linked to treatment from systematic variation caused by baseline cultivar differences, thereby facilitating the identification of metabolites responsive to pathogens. The OPLS-DA score plots (**Supplementary Figure 1**) showed minimal separation between control and pathogen-inoculated samples at 24 hours post-inoculation for Sifra, consistent with the limited early metabolic response noted in the PLS-DA analysis. At 48 hours, however, clear separation emerged in both cultivars, with Valor showing the most significant distinction between treatment groups along the predictive component. Sifra also exhibited separation at this time point, though it was less pronounced than Valor’s. The predictive component captured pathogen-induced metabolic variation, while the orthogonal component filtered out systematic cultivar-specific baseline differences. By 72 hours, the trend reversed, with Sifra demonstrating greater separation between control and pathogen-stressed samples, while Valor’s discrimination weakened significantly compared to the 48-hour mark, reflecting a temporal shift in defense strategies between the two cultivars.

### 3.2 Heatmap-based metabolic changes in pathogen-stressed and control potato cultivars

**Figure 4:**
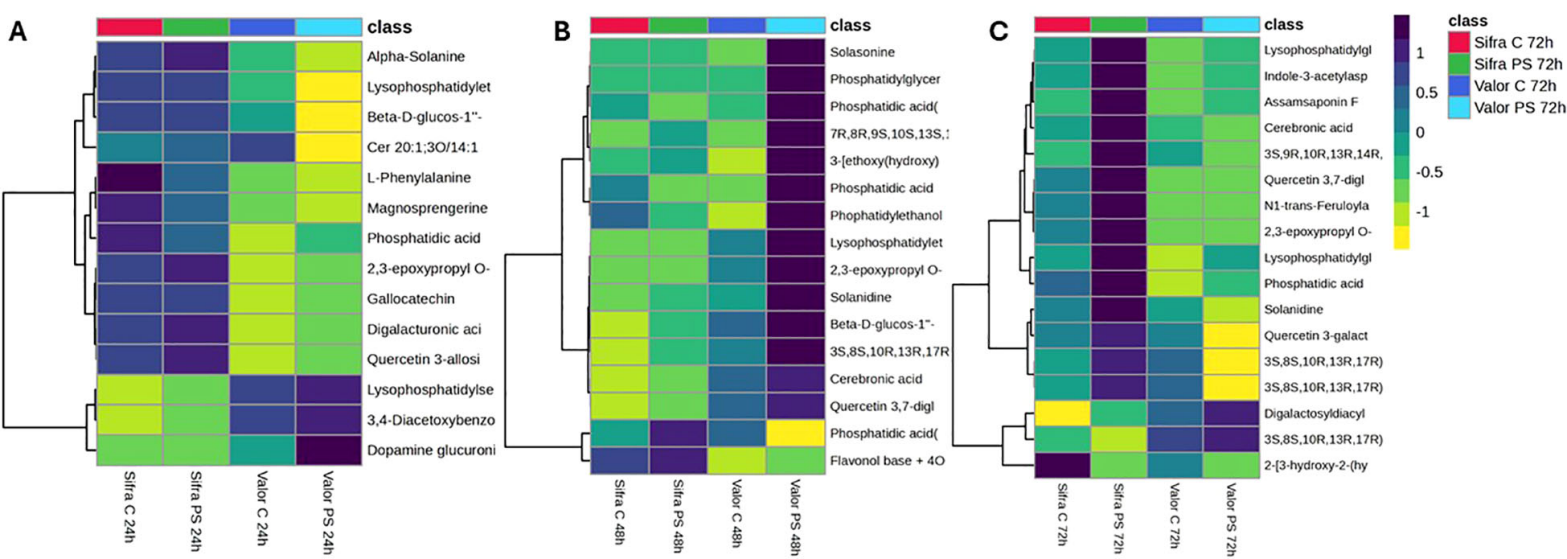
Heatmap of differentially accumulated metabolites in the Sifra and Valor potato cultivars under control **(C)** and pathogen stress (PS) conditions at 24 h **(A)**, 48 h **(B)**, and 72 h **(C)** post-infection. The heatmap illustrates the relative abundance of VIP-selected metabolites (VIP > 1), with purple indicating upregulation (+1) and yellow indicating downregulation (–1). Sifra samples showed early and sustained metabolite upregulation across all time points. In contrast, Valor displayed low metabolite levels at 24 h, a strong induction at 48 h, and a partial response at 72 (h) Clustering highlights distinct metabolic profiles that are dependent on both genotype and time in response to pathogen stress.

Heatmap analysis was employed to examine metabolite changes in Sifra and Valor potato cultivars at 24, 48, and 72 hours post-inoculation to investigate how the different potato varieties respond to pathogen infection. The heatmap displayed metabolite levels on a colour scale ranging from 1 (yellow, downregulation) to +1 (navy black, upregulation). This analysis facilitated identifying how metabolite patterns shift in response to pathogen attack (**Figures 4A–C**) (**Supplementary Tables 1–3**). The heatmap differentiation was based on a few metabolites, 14 for 24 hours, 16 for 48 hours and 17 for the 72 hour interval. The metabolites selected contribute towards the heatmap had the highest VIP scores (**Supplementary Tables 4A–C**). Overall, and based on the selected metabolites, the *P. syringae*-induced differentiation was only evident at 24 and 48 hours post-inoculation.

#### 3.2.1 High basal metabolism in Sifra and weak early activity in valor at 24 hours

At 24 hours post-infection, Sifra exhibited a strong upregulation of several metabolites in both control and pathogen-inoculated plants. Metabolites such as alpha-solanine, lysophosphatidylethanolamine, beta-D-glucoside, ceramide, l-phenylalanine, magnosprengerine, phosphatidic acid, 2, 3-epoxypropyl O-galactopyranosyl(1-6)galactopyranoside, gallocatechin, digalacturonic acid, and quercetin 3-alloside, were upregulated in Sifra samples. The upregulation of these metabolites was non-discriminatory between the inoculated and uninoculated sets; it was therefore assumed that Sifra’s early metabolic profile is primarily influenced by high basal activity rather than a targeted response to infection.

In contrast, Valor demonstrated downregulation of most of the same metabolites during this early stage of 24 hours post-inoculation with *P. syringae*. Control plants of Valor had lower basal metabolite levels, while pathogen-inoculated Valor plants exhibited mixed responses, showing upregulation of Lysophosphatidylserine, 3, 4-Diacetoxybenzoicacid and dopamine glucuronide.

#### 3.2.2 Emerging defence response in Sifra and peak response in valor at 48 hours

At 48 hours, Valor exhibited its clearest and strongest metabolic response. Pathogen-inoculated plants showed significant increases in solasonine, solanidine, phosphatidylglycerol, phosphatidic acid, phophatidylethanolamine, lysophosphatidylethanolamine, cerebronic acid, and Quercetin 3, 7-diglucoside, which were mildly upregulated in Valor’s control samples. Since the metabolomic responses were differentiated between the pathogen-inoculated and uninoculated sets, this indicated a marked defence response. The 48-hour mark represented the peak of Valor’s metabolic activation.

In contrast, Sifra demonstrated more selective metabolic changes compared to its 24-hour profile. At 48 hours post-inoculation, Sifra flattened with just mild upregulation and downregulation, and with very little differentiation between the inoculated and uninoculated treatments. Although the number of significantly upregulated metabolites was lower than that of Valor, Sifra’s pathogen-inoculated plants still exhibited slightly elevated levels of defence-related compounds such as solasonine, solanidine and upregulation of flavanol derivative compounds.

#### 3.2.3 Ongoing response in Sifra and declining activity in valor at 72 hours

By 72 hours, Sifra induced the production of several defence-related metabolites, including phosphatidic acid, lysophosphatidylglycerol, digalactosyldiacylglycerol, quercetin 3, 7-diglucoside, quercetin 3-galactoside and solanadine. These changes indicated a continued metabolic adjustment in response to infection. In contrast, Valor had flattened at 72 hours post-inoculation. While some metabolites, such as digalactosyldiacylglycerol and solanidine remained upregulated in Valor, the overall pattern was irregular and marked a significant shift from 48 hours. Compared to the sharp response observed at 48 hours, the 72-hour profile appeared more diffuse and less coordinated, indicating a decline or loss of momentum in its defence response. Work by Dahlin et al. (2017) and Mierziak et al. (2014) proved that solanine, solanidine and flavanol derivative compounds were proven to be defense related compounds.

#### 3.2.4 Metabolite changes over time after treatment

**Figure 5:**
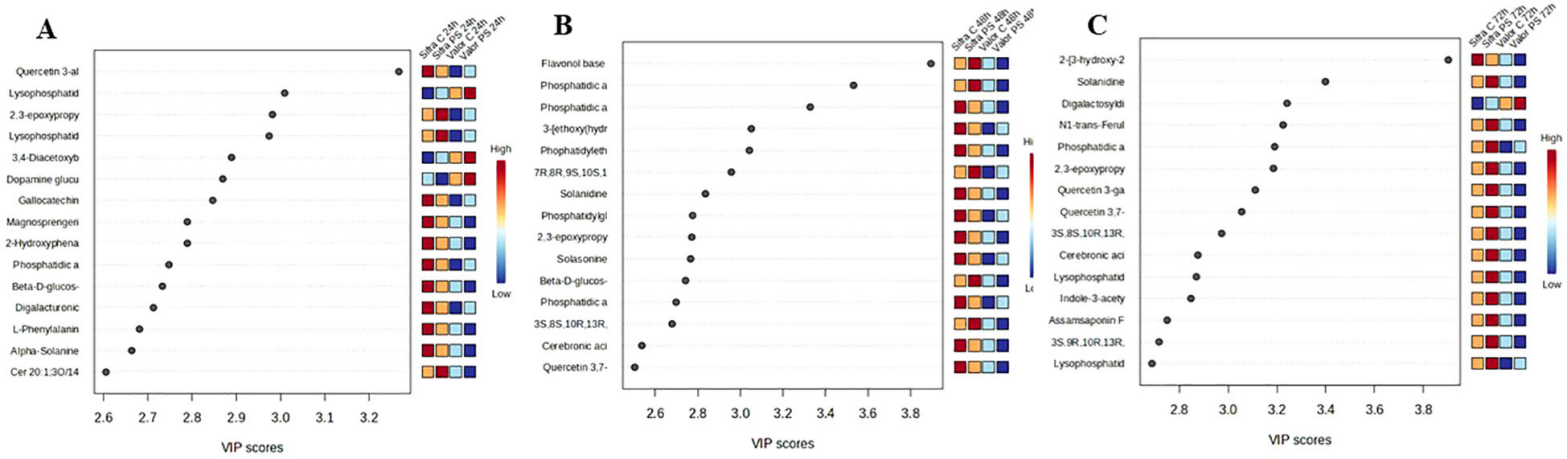
Top 15 discriminatory metabolites at 24, 48, and 72 hours post-treatment. Panels **(A–C)** display VIP plots and heatmaps from PLS-DA, illustrating the key metabolites that distinguish the treatment groups at each time point: 24 hours **(A)**, 48 hours **(B)**, and 72 hours **(C)**. Each time point presents a unique metabolite profile, with glycoalkaloids consistently featured, suggesting a sustained defence-related response.

The metabolite profiles recorded at 24, 48, and 72 hours post-treatment showed significant changes over time. Using Partial least squares discriminant analysis (PLS-DA), the 15 most up- and down-regulated metabolites were identified at each time point based on their variable importance in projection (VIP) scores. Only metabolites with a VIP score exceeding 1 were selected. These findings were illustrated through VIP plots and hierarchical clustering heatmaps, which highlighted the key metabolites that effectively distinguished between treatment groups and their levels across various treatment conditions (**Figures 5A–C**).

At 24 hours, the early response involved lipid signalling, with quercetin 3-alloside having the highest VIP score of 3.4, followed by several lysophospholipids and other compounds associated with antioxidant activity and membrane signalling, including dopamine glucuronide, gallocatechin, and magnosprengerin. These metabolites were particularly effective in separating *P. syringae*-treated samples from their uninoculated controls, suggesting the early activation of oxidative stress responses and signalling pathways. Alpha-solanine, a glycoalkaloid recognised for its role in plant defence (Liu et al., 2024), also ranked among the top contributors, indicating the initiation of specialised defence-related metabolism.

At 48 hours, lipid and glycoalkaloid accumulation characterised the response, with a different set of metabolites dominating the profile, including flavonol base compounds, phosphatidic acids, and glycoalkaloids such as solanine and solasonine. These metabolites are associated with alterations in membrane lipid composition and defence-related pathways (Winkiel et al., 2023).

A stronger defence-related activity was observed at 72 hours. The metabolic profile shifted yet again, with prominent metabolites including solanidine, solanine, digalactosyldiacylglycerol, and feruloyl-containing compounds. These compounds play a role in plant defence and stress-related signalling (Gao et al., 2014; Liu et al., 2024).

The metabolites varied significantly across the three time points, indicating that different metabolic pathways were activated at each stage. For instance, quercetin 3-alloside and lysophospholipids were upregulated at 24 hours but did not rank among the top separators at the subsequent time points of 48 hours and 72 hours. At 48 hours, key contributors included flavonol-based compounds, phosphatidic acids, and glycoalkaloids such as solanine and solasonine. These metabolites were upregulated with VIP scores between 3.5 and 3.9. By 72 hours, the main metabolites shifted to solanidine, feruloyl conjugates, and indole-related compounds with a VIP score between 3.4 and 3.8. Some metabolites, like phosphatidic acid compounds, appeared at multiple time points; however, no single metabolite consistently ranked in the top positions across all three time points, suggesting a time-dependent metabolic response to treatment.

At the chemical class level, glycoalkaloids and related steroidal alkaloids were consistently present across all time points. Alpha-solanine was observed at 24 hours, while solanine and solasonine were prominent at 48 hours. At 72 hours, solanidine and solanine again ranked among the top contributors. Although the individual compounds varied, the consistent presence of glycoalkaloids indicates that their production is a sustained and central feature of the treatment response. These compounds are commonly associated with antimicrobial activity, stress tolerance, and overall defence mechanisms in plants (Liu et al., 2024).

### 3.3 Pathway analysis: temporal metabolic reprogramming in response to infection

To better understand the observed metabolite changes, pathway enrichment and topology analyses were conducted using MetaboAnalyst’s MetPA tool across all three time points (24 h, 48 h, and 72 h post-inoculation). This approach helped in placing the differentially accumulated metabolites (VIP > 1) within broader biological pathways, as defined in the KEGG database. The resulting enrichment scores and pathway impact values gave an insight into the timing and nature of the metabolic responses in each cultivar (**Supplementary Figures 2 A–C**, **Supplementary Table 4A–C**).

#### 3.3.1 24 h Post-inoculation: early activation of secondary and signalling pathways

At 24 hours post-inoculation, pathway analysis revealed significant activation of secondary metabolism and membrane-associated signalling processes. The most enriched pathways included the biosynthesis of tropane, piperidine, and pyridine alkaloids, ether lipid metabolism, flavone and flavonol biosynthesis, and phenylalanine metabolism. Notably, phenylalanine metabolism exhibited the highest pathway impact (0.423), indicating its central role in the early defence response by supplying precursors for phenolic compounds and flavonoids (**Supplementary Tables 4A–C**).

At 24 hours, Sifra exhibited elevated levels of defence-related metabolites, including quercetin 3-alloside, alpha-solanine, and dopamine glucuronide. However, these increases were observed in both the control and *P. syringae*-treated plants, indicating that the response was likely part of Sifra’s natural (basal) metabolism rather than a direct reaction to the pathogen. The enrichment of ether lipid metabolism at this stage may suggest early preparations for membrane changes or signalling. In contrast, Valor demonstrated a smaller yet more distinct increase in specific metabolites only after *P. syringae* infection, indicating that Valor responded to the pathogen sooner than Sifra.

#### 3.3.2 48 h post-inoculation: peak lipid remodelling and delayed response in valor

By 48 hours, a significant shift was observed in metabolic regulation. Glycerophospholipid metabolism emerged as the most enriched and biologically impactful pathway impact (0.246), indicating active membrane lipid turnover and signal transduction. This finding aligns well with the PLS-DA and heatmap data, which revealed a dramatic upregulation of phosphatidylglycerol, phosphatidic acid, and solanidine in the Valor cultivar. This suggests that Valor mounts a delayed yet intense metabolic response current point (**Supplementary Tables 4A–C**).

Other enriched pathways included ether lipid metabolism and glycerolipid metabolism, underscoring the crucial roles of lipid signalling and structural adaptation during mid-stage infection. Notably, Sifra maintained elevated levels of key defence metabolites at this stage but exhibited more moderate shifts in pathway activity, consistent with its early response and stable metabolic regulation.

#### 3.3.3 72 h post-inoculation: sustained defence via glycerolipid and secondary metabolism

At the 72-hour mark, the metabolic focus shifted towards sustained defence and structural maintenance. Glycerolipid metabolism remained the most statistically enriched pathway, while the biosynthesis of various plant secondary metabolites exhibited a notably high pathway impact (0.24). These findings indicate continued investment in both lipid homeostasis and the production of specialised metabolites, including antimicrobial and antioxidant compounds (**Supplementary Tables 4A–C**).

Sifra consistently demonstrated upregulation of defence-related compounds such as lysophosphatidylglycerol, indole-3-acetylaspartate, and assamsaponin F, reflecting a sustained and coordinated response. In contrast, Valor displayed a more erratic pattern, with some secondary metabolite upregulation but lower consistency across metabolic classes. This deviation further supports the notion of metabolic preparedness in Sifra compared to a reactive and less efficient defence in Valor.

## 4 Discussion

This study employed an untargeted LC-MS metabolomics to investigate dynamic metabolic reprogramming in contrasting potato cultivars, Sifra and Valor, following inoculation with *P. syringae*. Cultivars Sifra and Valor displayed genotype-specific metabolomic responses to inoculation with *P. syringae*. Additionally, these responses were time-dependent as they were differentiated at 24, 48 and 72 hours post-inoculation. A plethora of other studies attempted to delineate genotype responses to pathogen inoculation and all showed metabolomic reprogramming in response to the pathogen. Differences between the studies are the suites of metabolites involved however with some metabolites consistently detected in most studies. The consistently detected metabolites include metabolites involved in the phenylpropanoid pathway and other crucial defense processes.

Beyond the effects of pathogens, the clear separation of metabolic profiles by cultivar at all time points suggests that cultivar identity is a primary driver of the metabolic signature. Risoli et al. (2025) reported similar findings, showing that cultivar identity often accounts for a larger proportion of metabolic variance than pathogen infection or treatment effects, even in open-field conditions. These results support the idea that the distinct metabolic signatures observed in this study are primarily influenced by cultivar-specific baseline metabolic organization rather than by the pathogen alone.

This research reveals that plant defence mechanisms go beyond simply classifying cultivars as resistant or susceptible. It demonstrates that plants can implement various timing strategies in their defence against pathogens (Kumari et al., 2024; Mashabela et al., 2023). Our metabolomic analysis of two potato cultivars responding to *P. syringae* infection identifies two distinct strategies: Sifra displays “metabolic preparedness, “ characterised by increased levels of defence-related metabolites prior to infection, while Valor employs “reactive defence, “ mounting a rapid and strong metabolic response immediately upon pathogen detection, which peaks at 48 hours before declining by 72 hours. This difference in response timing aligns with the framework proposed by Risoli et al. (2025), which emphasizes that cultivar identity determines genotype-specific metabolic kinetics and response trajectories, often surpassing the direct impact of the stressor itself.

### 4.1 Metabolic preparedness vs reactive defence

Sifra exhibits higher levels of alpha-solanine, quercetin 3-alloside, and lysophospholipids in both control and treated plants at 24 hours post-inoculation (hpi), in contrast to Valor’s lower baseline. This observation may suggest an evolutionary strategy where the plant remains in a constant state of readiness, rather than waiting for a specific trigger to initiate its defence (Züst and Agrawal, 2017). This idea is consistent with the concept of systemic acquired resistance described by Fu and Dong (2013), which posits that signals from a local infection can prepare the entire plant for potential future attacks. Additionally, previous research has shown that plants significantly reprogram their metabolism in response to pathogens (Bednarek, 2012).

Our metabolomics data illustrate that the duration and timing of this metabolic reprogramming are as crucial as its intensity for effective defence. As shown in our PCA and PLS-DA analyses (**Figures 2**, **3**), these timing differences create distinct metabolic signatures that differentiate the two cultivars across all time points. In support of this, Kopczewski et al. (2022) found that cucumber plants infected with *P. syringae* exhibited metabolic changes not only at the infection site but throughout the entire plant, highlighting the importance of systemic metabolic responses in defence. Studies also emphasise that the timing of pathway induction is vital in determining resistance, particularly in long-term defence mechanisms involving phenylpropanoid and glycoalkaloid biosynthesis (Dong and Lin, 2021; Yadav et al., 2020; Liu et al., 2024).

### 4.2 Early response phase (24 hours): strong pathogen recognition in valor vs. elevated basal metabolism in Sifra

By 24 hours post-inoculation (hpi), Sifra and Valor exhibit fundamentally different metabolic baselines. As clearly shown in the heatmap analysis (**Figure 4A**), Sifra displays constitutively elevated levels of defence metabolites, akin to wild crop relatives that maintain ongoing baseline defences (Züst and Agrawal, 2017), even in the absence of infection. In contrast, Valor shows minimal accumulation of pre-induction metabolites, indicating a reliance on pathogen-induced activation of its defence mechanisms (Ahuja et al., 2012; Balmer et al., 2013). The early activation of phenylalanine metabolism in Sifra (pathway impact: 0.423) is noteworthy. The pathway enrichment analysis (**Supplementary Figure 2A**) confirms that this pathway produces various defence compounds, including flavonoids, lignin, and antimicrobial phenolics (Dixon and Paiva, 1995; Maeda and Dudareva, 2012; Vogt, 2010). Recent advances in synthetic biology have shown that genetic engineering can enhance these pathways, suggesting that continuous activation of these defences could improve crop resistance. In experiments involving rice and potato-virus interactions, early activation of the phenylpropanoid pathway has been linked to increased disease resistance (Das et al., 2025; Kogovšek et al., 2016). Additionally, the sustained activity of ether lipid metabolism in Sifra at this early stage supports findings that changes in membrane composition can act as an early defence barrier (Hou et al., 2016). This may explain why some cultivars exhibit resistance even without visible signs of induced defences, as they are already primed (Conrath et al., 2015).

### 4.3 Peak response phase (48 hours): delayed intensity vs continued stability

At 48 hpi, Sifra and Valor exhibit contrasting metabolic strategies (Balmer et al., 2013; Bigeard et al., 2015). The dramatic difference between cultivars is most apparent in (**Figure 4B**), where Valor undergoes early metabolic reprogramming, marked by a significant increase in glycerophospholipid metabolism (pathway impact: 0.246). The accumulation of phosphatidic acid and phosphatidylglycerol indicates active membrane remodeling and signalling (Testerink and Munnik, 2011; Ruelland et al., 2015; Hou et al., 2016). This spike likely reflects the activation of pattern-triggered and effector-triggered immune responses (Jones and Dangl, 2006; Bigeard et al., 2015). However, such a robust response may be energetically expensive, leading to an inability to maintain defence, as evidenced by a decline at 72 hpi (Berger et al., 2007; Walters et al., 2013). A similar decline in metabolic defence was observed in Valor, mirroring trends seen in other potato-virus pathosystems (Manasseh et al., 2023; Zheng et al., 2012). The increase in glycerophospholipids observed in Valor aligns with previous studies indicating that lipid-based signalling is crucial for regulating plant immune responses (Hou et al., 2016; Cacas et al., 2016). Phosphatidic acid, as a signalling molecule, is involved in activating defence gene expression and inducing structural changes in membranes during infection (Testerink and Munnik, 2011; Hong et al., 2010).

### 4.4 Sustained response phase (72 hours): maintenance vs decline

By 72 hpi, the ability to maintain defence over time is vital. (**Figure 4C**) demonstrates that Sifra continues to show elevated levels of solanidine and lysophosphatidylglycerol, indicating an ongoing defence response. In contrast, Valor shows a reduction in metabolic activity, which may stem from energy depletion or pathogen suppression (Berger et al., 2007; Bostock et al., 2014). Lee et al. (2013) demonstrated that extracellular metabolites produced by *P. syringae* can actively suppress plant defence responses. This finding may help explain the decline in Valor’s metabolic defences during the later stages of infection. Our research highlights that not only the presence but also the timing of these accumulations is critical for effective defence (Liu et al., 2024). The ongoing metabolism in Sifra at 72 hpi stands in contrast to what is typically observed in susceptible plants, where defence often fails due to pathogen interference or energy depletion (Dodds and Rathjen, 2010; Doehlemann et al., 2017). This ability to maintain a sustained response may be crucial for achieving durable resistance (Mauch-Mani et al., 2017; Conrath et al., 2015).

### 4.5 Key metabolite classes and their functional significance

Our VIP analysis (**Figures 5A–B**) identified several metabolite classes that serve as molecular signatures of the different defence strategies:

### 4.6 Flavonoids as early defence markers

The early and consistent presence of quercetin 3-alloside (VIP score: 3.4) in Sifra suggests a novel form of defence priming (Agati et al., 2012; Falcone Ferreyra et al., 2012). Quercetin compounds not only fight pathogens directly but also help activate other defence mechanisms (Treutter, 2006). The glycosylation of quercetin into quercetin 3-alloside may enhance its stability and facilitate storage within the cell (Vogt and Jones, 2000; Bowles et al., 2005). This finding is particularly relevant given recent advancements in our understanding of flavonoid biosynthesis and regulation (Nabavi et al., 2020; Tohge et al., 2017). These attributes position quercetin 3-alloside and other early markers, such as alpha-solanine, as promising candidates for resistance screening and breeding (Sakurai, 2022; Liu et al., 2024).

### 4.7 Glycoalkaloids: temporal defence architecture

We observed consistent trends in glycoalkaloid accumulation: alpha-solanine at 24 hours hpi, solanine and solasonine at 48 hpi, and solanidine at 72 hpi. This temporal progression is visible across the VIP plots in (**Figures 5A–C**). This pattern indicates a strict regulation of glycoalkaloid metabolism over time (Friedman, 2006; Zhao et al., 2021). Each of these compounds may influence different aspects of the pathogen’s biology (Liu et al., 2024). The shift from glycosylated molecules like alpha-solanine to the aglycone solanidine suggests that enzymatic modifications play a role in modulating the activity and distribution of these molecules during the defence response (Itkin et al., 2013). Shenkarev et al. (2007) explained these key enzyme steps in potato glycoalkaloid production, showing how the plant regulates its defence chemicals. Glycosylated molecules may also function as stable reservoirs, readily activated when needed (Cardenas et al., 2015). Research has shown that glycoalkaloid biosynthesis is regulated at both the gene expression and post-transcriptional levels, with specific transcription factors directly involved in activating the key biosynthetic pathway (Liu et al., 2024; Itkin et al., 2013). Multiple genetic controls, including networks of co-expressed genes, regulate the metabolism of steroidal glycoalkaloids in potato tubers. This supports the notion that the differences between cultivars, such as Sifra and Valor, stem from the genetic regulation of these pathways (Shen et al., 2021). The consistent activity of this pathway in Sifra may stem from variations in the regulation of these genes.

Dastmalchi et al. (2019) showed that glycoalkaloid content infection in potato varies among different cultivars. Notably, cultivars with higher glycoalkaloid levels also demonstrated greater antibacterial activity, suggesting a direct relationship between metabolite accumulation and the intensity of the plant’s defence. Similarly, Dahlin et al. (2017) showed that glycoalkaloids, such as α-solanine and solanidine, can destabilise cell membranes and internal structures of *Phytophthora infestans*, exhibiting broad antimicrobial properties. Together, these findings support the idea that Sifra’s early and sustained glycoalkaloid accumulation may provide a stronger and more durable biochemical defence against pathogens like *P. syringae.*

### 4.8 Lipid signalling: membrane dynamics and communication

Our findings reveal high concentrations of lysophospholipids and phosphatidic acid derivatives, underscoring the importance of lipid signalling in plant defence (Hou et al., 2016; Ruelland et al., 2015). Sifra consistently exhibits elevated levels of lysophosphatidylcholine and lysophosphatidylethanolamine, both of which function as membrane components and signalling molecules (Cacas et al., 2016; Hou et al., 2016). Their persistent presence in Sifra suggests that lipid signalling is continuously active, potentially through phospholipase A activity, which may prepare cell membranes to transmit signals swiftly upon pathogen detection (Hong et al., 2010). Pathway impact and topology analysis indicate that glycerophospholipid metabolism occupies a central position within the defence-related metabolic network, functioning as a regulatory hub integrating membrane dynamics with downstream defence responses. This systems-level role of lipid pathways mirrors the network-based interpretation proposed by Risoli et al. (2025).

### 4.9 Evolutionary and agricultural implications

The contrasting strategies of Sifra (constitutive defence) and Valor (induced defence) illustrate evolutionary trade-offs in resource allocation (Züst and Agrawal, 2017). While constant defences can deplete energy resources for growth and reproduction (Stamp, 2003; Koricheva, 2002), pathogen-induced responses conserve resources but may result in delayed protection (Karban and Baldwin, 1997; Agrawal, 1999). In high-disease environments, the reliability of Sifra’s constitutive defences is crucial (Walters et al., 2013; Mauch-Mani et al., 2017), especially as climate change intensifies disease pressures (Bebber et al., 2013; Ghini et al., 2016; Velásquez et al., 2018).

### 4.10 Applications for crop improvement

The key pathways and metabolites identified in our study offer promising targets for engineering disease resistance (Pollastri and Tattini, 2011). Enhancing phenylalanine metabolism, flavonoid biosynthesis, and glycoalkaloid biosynthesis could bolster resistance in susceptible cultivars. Recent advancements in synthetic biology show that it is possible to engineer complex defence pathways in plants (Nielsen and Keasling, 2016). Engineering transcription factors may allow us to replicate the coordinated defences observed in Sifra (Chattopadhyay et al., 2019). The time-course metabolite patterns identified in this study could serve as biomarkers for resistance breeding (Ghatak et al., 2018). Early markers, such as quercetin 3-alloside and alpha-solanine, could facilitate the rapid identification of resistant lines, while sustained responses at 72 hpi may indicate durable resistance (Sakurai, 2022; Mur et al., 2017). This strategy aligns with recent metabolomics-driven approaches in cereals and Solanaceae crops (Effah et al., 2023; Jose et al., 2023).

## 5 Conclusion

This time-course metabolomics study demonstrates that different potato cultivars show distinct temporal patterns in their response to *P. syringae* infection. The cultivar Sifra begins its defence metabolism early, at 24 hours post-infection, and maintains this response consistently up to 72 hours. In contrast, Valor exhibits a reactive pattern, with a slow but vigorous metabolic activation that peaks at 48 hours before declining. A key finding is that the timing and consistency of defence responses are more important than their intensity. Sifra rapidly activates all major defence-related pathways, such as phenylalanine metabolism, flavonoid biosynthesis, and ether lipid metabolism. This early activation results in a sustained build-up of essential defence metabolites, including quercetin 3-alloside, gallocatechin, alpha-solanine, and lysophospholipids, which together establish a coordinated and long-lasting protective response. Conversely, Valor shows a slower activation of glycerophospholipid and glycoalkaloid biosynthesis, which, although vigorous at 48 hours, is transient and less effective by 72 hours. These findings have practical implications for potato breeding. The early and sustained build-up of quercetin 3-alloside and alpha-solanine can serve as biomarkers for high-throughput screening of resistant cultivars. Additionally, the prolonged induction of flavonoid, glycoalkaloid, and lipid metabolism pathways presents a promising target for enhancing resistance. Temporal metabolite profiling is an effective tool for identifying traits associated with durable defence. Collectively, these metabolic signatures offer actionable targets for breeding potato varieties with improved resistance to *P. syringae* and potentially other bacterial pathogens, contributing to more durable and sustainable potato production systems.

## Data Availability

The original contributions presented in the study are included in the article/**Supplementary Material**. Further inquiries can be directed to the corresponding author.
