# Supplementary material for: A time-resolved metabolomics study of potato cultivar responses to Pseudomonas syringae
Source: Front Plant Sci. 2026 Feb 11;17:1760708. doi: 10.3389/fpls.2026.1760708 (PMC12934511; doi:10.3389/fpls.2026.1760708)

Supplementary Material

##
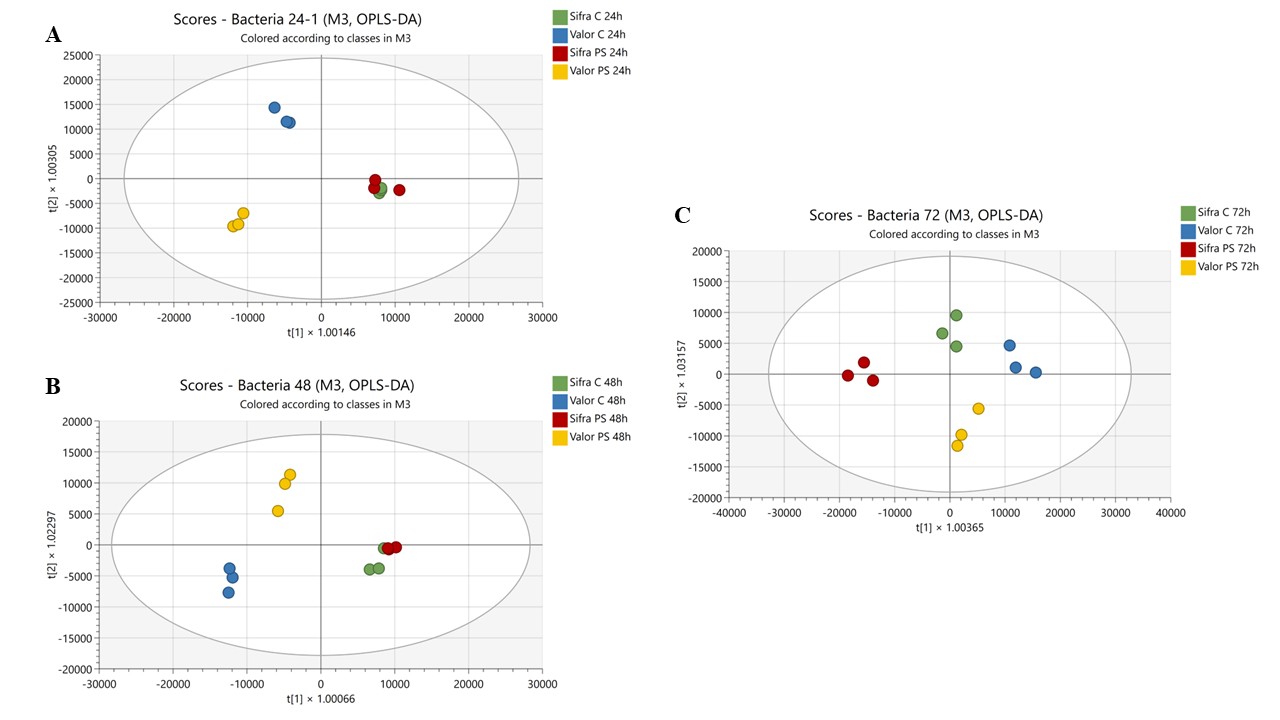
Supplementary Figures

**Supplementary Figure 1 (A-C).** Orthogonal partial least squares discriminant analysis OPLS-DA score plots of potato cultivar metabolic responses to *Pseudomonas syringae* infection across three time points. OPLS-DA score plots showing the separation between control (C) and pathogen-stressed (PS) samples for Sifra and Valor cultivars at 24 hours, 48 hours, and 72 hours post-inoculation


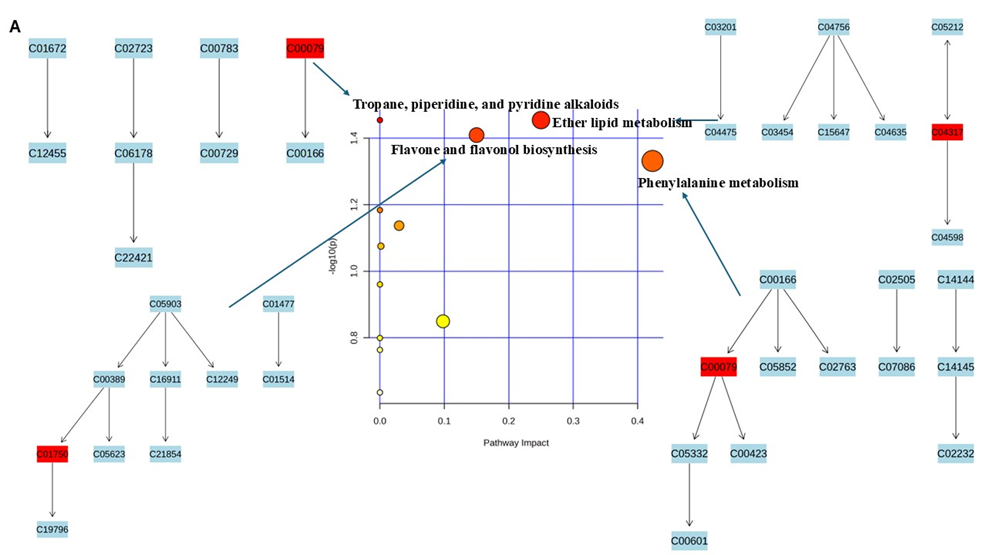


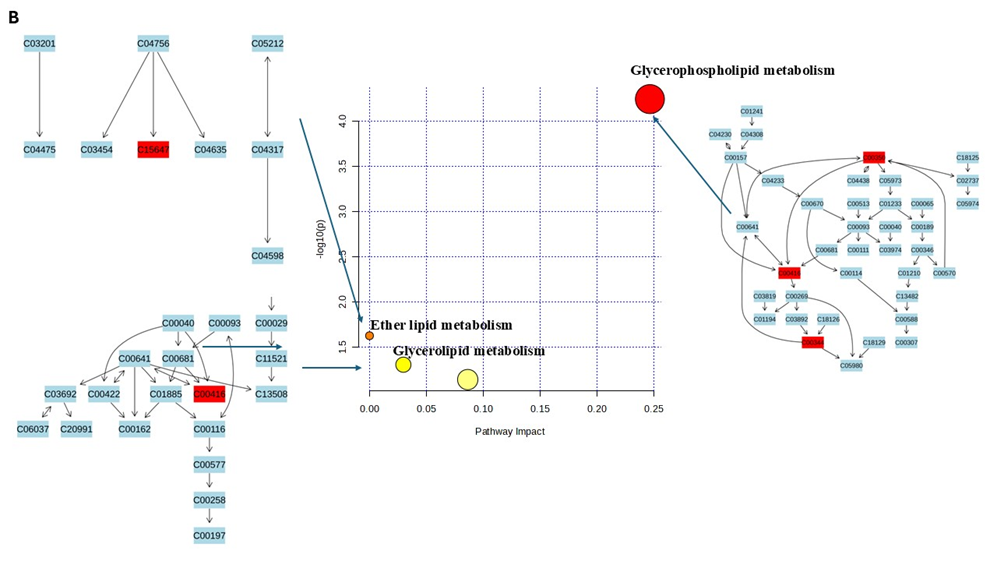


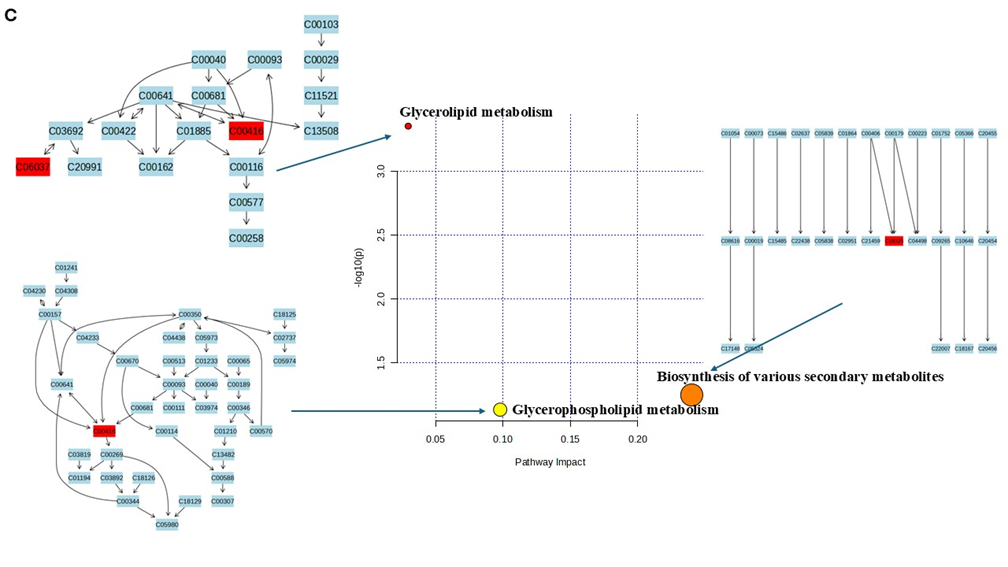


**Supplementary figure 2 (A-C**). Pathway enrichment and topology analysis of differential metabolites at 24, 48, and 72 h post-inoculation. Bubble plots show enriched metabolic pathways based on VIP > 1 metabolites for each time point. The x-axis indicates pathway impact; the y-axis shows –log₁₀(p) for enrichment significance. Bubble size and colour reflect pathway relevance. At 24 h **A**, Sifra showed early activation of phenylalanine, flavonoid, and alkaloid pathways. At 48 h **B**, Valor exhibited delayed glycerophospholipid and ether lipid metabolism enrichment. By 72 h **C**, Sifra sustained defence-related pathway activity, while Valor's metabolic response weakened.

## Supplementary Tables

**Table 1**. Principal metabolites associated with *P. syringae* 24 hours post inoculation, annotated with LC-MS data (Excel file)

**Table 2**. Principal metabolites associated with *P. syringae* 48 hours post inoculation, annotated with LC-MS data (Excel file)

**Table 3.** Principal metabolites associated with *P. syringae* 72 hours post inoculation, annotated with LC-MS data (Excel file)

**Table 4.** (A-C) Summary of enriched metabolic pathways at 24, 48, and 72 hours post-inoculation based on differentially accumulated metabolites (VIP > 1). Pathway analysis was performed using MetaboAnalyst and KEGG database annotations. Match status indicates the number of significant metabolites mapped per pathway. P-values, adjusted p-values (Holm), false discovery rate (FDR), and pathway impact scores are shown. At 24 h, enrichment of phenylalanine metabolism, flavonoid biosynthesis, alkaloid, and ether lipid metabolism reflects early activation of secondary and lipid-based defences in the resistant cultivar Sifra. At 48 h, Valor exhibited delayed but strong enrichment of glycerophospholipid and ether lipid pathways, indicating late onset of membrane-related signalling. At 72 h, Sifra sustained defence activation via glycerolipid metabolism and secondary metabolite biosynthesis, while Valor showed reduced pathway engagement.

**
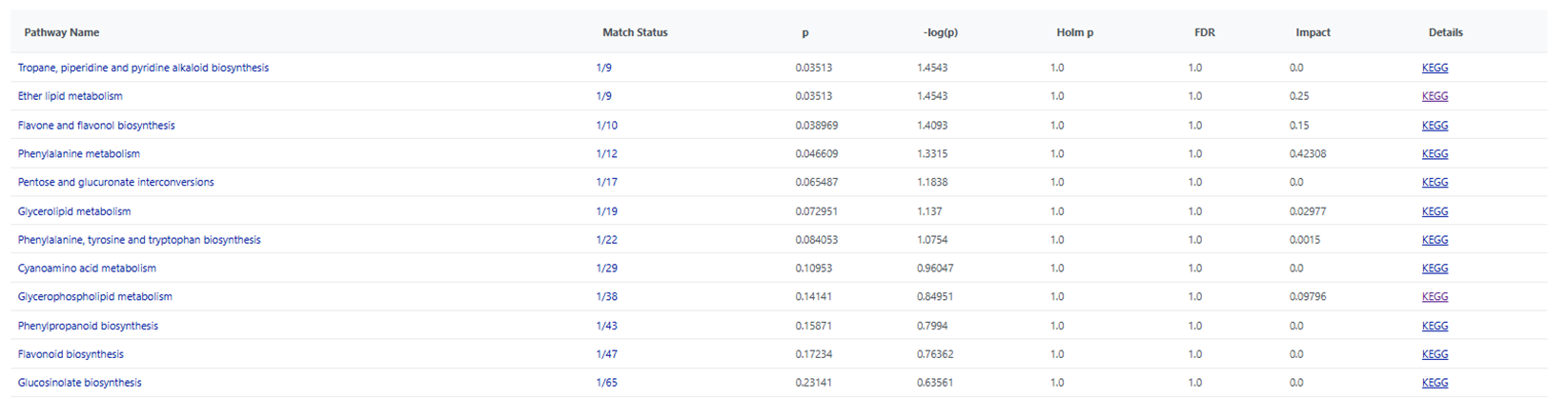
Table 4A**

**
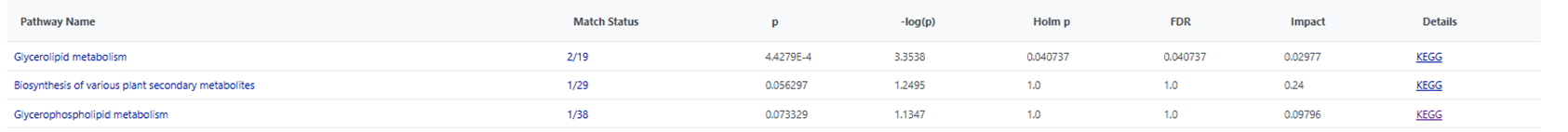
Table 4B**

**Table 4C**


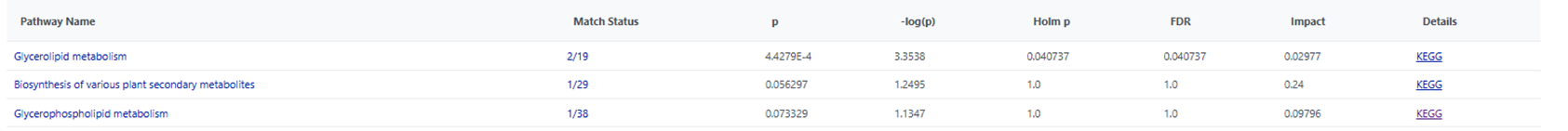

Supplement: Supplementary file 1 [file DataSheet1.docx]
